# Therapeutic promise of engineered nonsense suppressor tRNAs

**DOI:** 10.1002/wrna.1641

**Published:** 2021-02-10

**Authors:** Joseph J. Porter, Christina S. Heil, John D. Lueck

**Affiliations:** ^1^ Department of Pharmacology and Physiology University of Rochester Medical Center Rochester New York USA; ^2^ Department of Neurology University of Rochester Medical Center Rochester New York USA

**Keywords:** gene expression, nonsense mutation, nonsense suppression therapy, nonsense suppressor tRNA, premature termination codon mutations, readthrough, translation

## Abstract

Nonsense mutations change an amino acid codon to a premature termination codon (PTC) generally through a single‐nucleotide substitution. The generation of a PTC results in a defective truncated protein and often in severe forms of disease. Because of the exceedingly high prevalence of nonsense‐associated diseases and a unifying mechanism, there has been a concerted effort to identify PTC therapeutics. Most clinical trials for PTC therapeutics have been conducted with small molecules that promote PTC read through and incorporation of a near‐cognate amino acid. However, there is a need for PTC suppression agents that recode PTCs with the correct amino acid while being applicable to PTC mutations in many different genomic landscapes. With these characteristics, a single therapeutic will be able to treat several disease‐causing PTCs. In this review, we will focus on the use of nonsense suppression technologies, in particular, suppressor tRNAs (sup‐tRNAs), as possible therapeutics for correcting PTCs. Sup‐tRNAs have many attractive qualities as possible therapeutic agents although there are knowledge gaps on their function in mammalian cells and technical hurdles that need to be overcome before their promise is realized.

This article is categorized under:

RNA Processing > tRNA Processing
Translation > Translation Regulation

## Graphical abstract

Nonsense suppressor tRNAs display promise as therapeutic agents for suppressing premature termination codons, and alleviating disease through seamless rescue of full‐length protein and stabilized mRNA.

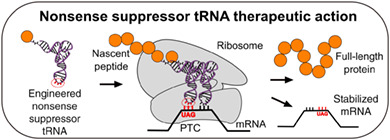

## 1 INTRODUCTION

**FIGURE 1:**
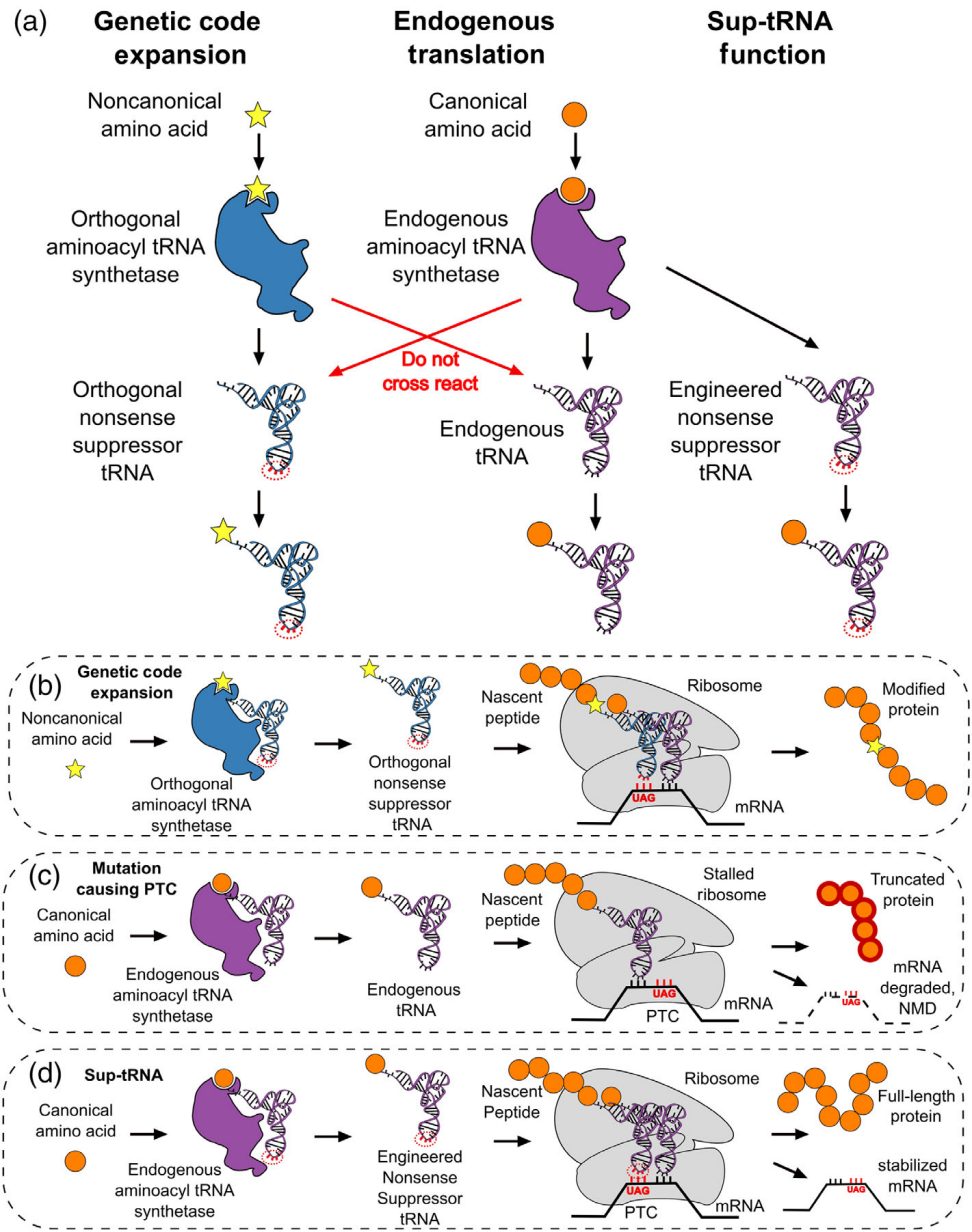
The applications of sup‐tRNAs and their function in translation. (a) Genetic code expansion involves the codon specific incorporation of a noncanonical amino acid (ncAA) into proteins in vivo. This is accomplished by introducing an orthogonal aminoacyl‐tRNA synthetase (o‐aaRS) specific for both the ncAA and its cognate orthogonal tRNA (o‐tRNA). This newly introduced pair is “orthogonal” as it does not cross‐react with any of the endogenous tRNAs or aaRSs. For genetic code expansion, the o‐tRNA most often is engineered as an amber sup‐tRNA to ensure site‐specific ncAA incorporation. In contrast, ACE‐tRNAs for therapeutic suppression of PTCs are designed to cross‐react with endogenous aaRSs while altering the anticodon to suppress a nonsense codon. (b) Genetic code expansion enables site‐specific incorporation of an ncAA by employing an o‐aaRS/amber nonsense suppressor o‐tRNA pair to suppress an amber stop codon in the mRNA of a gene of interest using cellular translation. (c) Premature termination codons (PTCs) arise from single nucleotide mutations that convert a canonical triplet nucleotide codon into a stop codon. The generation of a PTC in an mRNA causes the translating ribosome to stop, resulting in the release of a truncated protein and mRNA degradation by nonsense‐mediated decay (NMD). (d) in a manner analogous to genetic code expansion, sup‐tRNAs are charged by an endogenous aaRS leading to PTC suppression on the ribosome, seamless rescue of full‐length protein expression, and stabilization of mRNA by inhibiting NMD

The genetic code is composed of nucleotide triplets called codons, which specify the amino acid added during protein synthesis. Of the 64 possible combinations of nucleotides making up codons, 61 code for the incorporation of an amino acid into the growing polypeptide chain on the translating ribosome, while three (UAA, UAG, and UGA) are reserved to signal for translation termination. A mutation (generally a single‐nucleotide substitution) that converts a codon originally coding for an amino acid to one of the termination codons is termed a nonsense mutation and results in a premature termination codon (PTC) in the protein‐coding sequence. Translation terminates prematurely at the PTC, producing a truncated protein which is nonfunctional or even harmful to the cell (Figure 1(c)) (J. T. Mendell & Dietz, 2001). Nonsense mutations account for 10%–15% of all genetic lesions leading to disease, causing nearly 1000 serious genetic disorders (Mort et al., 2008). Due to the high prevalence and unifying mechanism of nonsense mutations resulting in disease, there have been significant efforts to identify PTC therapeutics.

Many of the past efforts to produce a PTC therapeutics have focused on nonsense suppression where an amino acid carried by a tRNA is incorporated into the nascent polypeptide chain at the PTC instead of premature translation termination and peptide release. Due to the deleterious role of nonsense mutations in gene expression and disease, nonsense suppression is an attractive strategy to prevent premature translation stop. Several approaches to suppress nonsense mutations have been pursued including small molecule readthrough agents like gentamicin and ataluren, which bind to the decoding center of the ribosome and alter its translation fidelity, allowing suppression of PTCs by near‐cognate aminoacyl‐tRNAs (Fan‐Minogue & Bedwell, 2008). Several nucleotide‐based strategies including targeted pseudouridylation of PTCs in mRNA, CRISPR/Cas9 gene editing and nonsense suppressor tRNAs (sup‐tRNAs) have been pursued as more specific methods to rescue PTC‐containing gene function (Karijolich & Yu, 2014; Krishnamurthy et al., 2019; Lueck et al., 2019). The sup‐tRNA approach makes use of anticodon‐engineered sup‐tRNAs (ACE‐tRNAs) designed to cross‐react with endogenous aminoacyl‐tRNA synthetases (aaRSs) for charging with the correct amino acid, while altering the anticodon for therapeutic suppression of PTCs and seamless rescue of full‐length protein expression.

In this review, we focus on the therapeutic promise of sup‐tRNAs while providing the historical context for the role of sup‐tRNAs as tools to understand biology. We will also provide background on the biogenesis of mammalian tRNAs as a treatment of this topic is germane to the use of sup‐tRNAs as therapeutics. Finally, we will discuss some of the concerns associated with sup‐tRNAs, possible routes of sup‐tRNA delivery, and compare the capabilities of sup‐tRNAs with other PTC therapeutics.

## 2 PTC THERAPEUTIC TECHNOLOGIES

To highlight all efforts being made to address PTC‐related disease, we are briefly treating the nature of PTC therapeutic technologies that are being investigated aside of sup‐tRNAs. In the concerted effort to identify PTC therapeutics, all possibilities should be exploited. Every different approach has its own advantages and disadvantages and all have their raison d'être. Most likely there will not be the one right approach but hopefully many will lead to a successful therapeutic.

Aminoglycosides, a class of ribosome‐binding antibiotics, are known to promote nonsense readthrough (W. F. Anderson et al., 1965; Davies et al., 1964; Gorini & Kataja, 1964). They have been reported to act as nonsense readthrough agents for the treatment of cystic fibrosis and *mdx* mice (an animal model for Duchenne muscular dystrophy; Barton‐Davis et al., 1999; Bedwell et al., 1997; Howard et al., 1996). For a comprehensive review of aminoglycosides and related molecules as PTC therapeutics, we refer to these reviews (Keeling et al., 2014; Lee & Dougherty, 2012). Several aminoglycosides have been used in clinical trials with underwhelming performance. For their long‐term application in patients, a series of obstacles must be overcome: (i) Aminoglycosides reduce translational fidelity and increase misincorporation of near‐cognate amino acids (Fan‐Minogue & Bedwell, 2008). Readthrough of a PTC by aminoglycosides can insert not only the original amino acid, but may also insert a different amino acid at a nonsense mutation. Over many rounds of translation, this will result in the production of several different protein variants, the sequence of which depends on the PTC identity and the readthrough agent used. Amino acid variation may affect proper protein folding, trafficking, and function and can have substantial effects on cellular processes. (ii) A common problem with nonsense suppression therapy is the possibility of natural termination codon (NTC) readthrough. In fact, genome‐wide NTC readthrough stimulated by the aminoglycoside G418 in vivo disrupts several biological processes with detrimental cellular effects (Wangen & Green, 2020). (iii) Aminoglycosides bind to the decoding center of the ribosome and at sufficient doses inhibit protein synthesis (Francois et al., 2005). Although these aminoglycoside antibiotics predominantly bind to bacterial ribosomes (Lynch & Puglisi, 2001), they can, to some extent, also bind to eukaryotic ribosomes, especially to mitochondrial ribosomes with severe effects (Hobbie et al., 2008; Qian & Guan, 2009). (iv) Furthermore, aminoglycosides pose the risk of strong oto‐ and nephrotoxicity, as previously reviewed (Dabrowski et al., 2018).

Recent efforts have focused on screening small libraries for identification of non‐aminoglycoside small molecule PTC readthrough (for a review, see [Keeling et al., 2014]). One candidate worth mentioning is PTC124 (also known as Ataluren or Translarna), a low molecular weight compound discovered in a high‐throughput screen by PTC Therapeutics Inc. (Welch et al., 2007). Despite low in vivo efficacy, PTC124 has been approved in the European Union for ambulatory patients aged 2 years and older with Duchenne muscular dystrophy resulting from nonsense mutations in the dystrophin gene. Recently, ELX‐02, a small molecule eukaryotic ribosomal selective glycoside, has shown promise as a PTC readthrough therapeutic (Crawford et al., 2020). Despite the impact on translational fidelity and readthrough of NTCs, small molecules that target the decoding center of the ribosome may possess a therapeutic window (Wangen & Green, 2020).

Another attractive new approach of nonsense‐suppression uses targeted pseudouridylation of nonsense codons (Karijolich & Yu, 2011; Morais et al., 2020). Pseudouridylation is a common, naturally occurring post‐transcriptional RNA modification in eukaryotic cells (McCown et al., 2020). The box H/ACA ribonucleoprotein (RNP) complex catalyzes the isomerization of a uridine residue (U) to pseudouridine (Ψ). In this approach, the first position of the nonsense codon, which is U for all three nonsense codons (UGA, UAG, and UAA), is converted into Ψ. This modification leads to the incorporation of near‐cognate amino acids (Fernandez et al., 2013), and thus to nonsense suppression. Site‐specific pseudouridylation of PTCs is achieved using an artificial H/ACA guide RNA, which is part of the box H/ACA RNP complex, targeting a specific PTC. Preliminary results of in vitro and in vivo application of targeted pseudouridylation show successful nonsense suppression (Karijolich & Yu, 2011), but efficient delivery and misincorporation pose obstacles for this approach. Unlike aminoglycosides or sup‐tRNAs, pseudouridylation raises little concerns about global NTC readthrough. On the downside, like aminoglycosides, pseudouridylation promotes misincorporation of near‐cognate amino acids. Similar to sup‐tRNAs, this technology can be delivered as RNA or DNA offering several delivery approaches. Unlike sup‐tRNAs, whose selectivity is only determined by the PTC identity, each pseudouridylation guide is tailored to a specific disease‐causing PTC. Therefore, sup‐tRNAs are closer to a one‐size‐fits‐all therapeutic for a given amino acid codon to PTC mutation, while targeted pseudouridylation is precision medicine.

There are several promising uses of CRISPR/Cas technologies for nonsense mutation intervention that includes prime editing, homologous recombination following double‐stranded breaks, and in‐frame deletions. However, there already exist several excellent reviews that cover the exciting possibilities of CRISPR/Cas (Knott & Doudna, 2018; Komor et al., 2017; Kotagama et al., 2019). It should be noted that the two approaches, targeted pseudouridylation, and CRISPR/Cas editing depend on the genomic context for targeting and therefore will confer no NTC readthrough, however, toxicity may occur through other off‐target mechanisms.

## 3 SUMMARY OF PTC SUPPRESSION IN DISEASE‐RELATED GENES USING SUP‐TRNAS

Sup‐tRNAs as a therapeutic approach to rescue protein function lost due to PTCs are not a completely new concept (Figure 1(d)). Several studies utilized sup‐tRNAs charged with amino acids and carrying a nonsense suppressing anticodon to address PTCs in certain disease‐related genes. However, this specific field of sup‐tRNA therapeutics remains at a modest number of publications up until now; all endeavors are shortly summarized in the following.

The first report of PTC suppression using sup‐tRNAs was in 1982, where Temple et al. restored β‐globin chain synthesis, which is deficient in β‐thalassemia (β^0^‐thalassemia), with a human amber (UAG) sup‐tRNA^Lys^ by microinjection in *Xenopus laevis* oocytes (Temple et al., 1982). Another early report by Panchal et al. showed suppression of a PTC in the Xeroderma pigmentosum group A (XPA) gene using a human opal (UGA) sup‐tRNA^Arg^ in Xeroderma pigmentosum patient‐derived cells (Panchal et al., 1999). Buvoli et al. (2000) presented the first in vivo report of PTC suppression in transgenic mice. They used a chloramphenicol acetyltransferase (CAT) reporter gene interrupted by an ochre (UAA) PTC and rescued CAT activity using a multicopy ochre (UAA) sup‐tRNA^Ser^ expression cDNA. Kiselev et al. provided another in vivo report, where they suppressed a PTC in the dystrophin gene of the mdx mouse, which is a model for Duchenne muscular dystrophy (Bulfield et al., 1984; Kiselev et al., 2002; Sicinski et al., 1989). In Ullrich disease, the collagen VI α2 gene carries a PTC caused by a frameshift mutation. Sako et al. (2006) used sup‐tRNA^Ser^ with four‐base anticodons to suppress this frameshift mutation in Ullrich disease fibroblasts and restored collagen VI expressions. Bordeira‐Carrico et al. (2014) provided the first report of full‐length protein expression and functional rescue of a human cancer‐associated gene using sup‐tRNAs in vitro. They restored full‐length E‐cadherin expression and function from a PTC interrupted CDH1 gene, which causes hereditary diffuse gastric cancer (HDGC) syndrome including lobular breast cancer (LBC), in mammalian cell lines using sup‐tRNA^Arg^. The most recent report of PTC suppression demonstrates the use of sup‐tRNAs for cystic fibrosis to restore full‐length cystic fibrosis transmembrane conductance regulator (CFTR) protein and restoration of PTC reporter activity in eukaryotic cells and mouse skeletal muscle (Lueck et al., 2019).

While these examples show the general feasibility of sup‐tRNAs to be utilized as possible PTC therapeutic, it is worthwhile to take a step back and look at the history of sup‐tRNA discovery and their application in genetic code expansion (GCE), a much more rapidly growing field optimizing this technology.

## 4 MAKING SENSE OF NONSENSE

Harnessing nonsense suppressor tRNAs has been entwined with our understanding of nonsense codons since the beginning. While 61 of the 64 possible nucleotide triplet codons had been discovered to code for specific amino acids, the three remaining were known as “nonsense” as they did not seem to code for anything (Brenner et al., 1965).

The first clues to unraveling the mystery of these nonsense codons were revealed by the work of several students at Caltech including Harris “Forever Amber” Bernstein. Harris was convinced to help pick thousands of mutant T4 bacteriophage plaques with the promise of naming the “amber” mutants after him (Stahl, 1995). The T4 bacteriophage amber mutants were nonfunctional in wild‐type (WT) bacteria strains, but phage function was restored by strains of bacteria carrying “amber suppressor genes” (Epstein et al., 2012). The observation that the nonsense codon introduced by the amber mutation resulted in polypeptide chain termination (Sarabhai et al., 1964), followed by careful reversion of mutant phenotypes with chemical mutagens, revealed that the “amber” mutants contained the UAG triplet. Later work showed that the “ochre” (following the color‐based naming convention) mutants contain the UAA triplet and that “nonsense” codons were more appropriately named “chain termination codons” (Brenner et al., 1965). Further work revealed the final “nonsense” codon to be the third termination codon, UGA or “opal” (Brenner et al., 1967; Sambrook et al., 1967; Zipser, 1967). The mechanism of nonsense suppression in the mutant bacteria was later shown to be the action of nonsense suppressor tRNAs (sup‐tRNAs) inserting a specific amino acid at the nonsense codon, allowing expression of full‐length protein in the phage (Capecchi & Gussin, 1965; Engelhardt et al., 1965; Goodman et al., 1968).

Following the discovery of sup‐tRNAs in prokaryotes, they were shown to be functional in a variety of eukaryotic systems including mouse tissue culture (Capecchi et al., 1975; Capecchi et al., 1977; Hudziak et al., 1982; Young et al., 1983), *Xenopus oocytes* (Bienz et al., 1980), *Drosophila* cell‐free extract (Kubli et al., 1982), yeast cell‐free extract (Tuite et al., 1983), monkey cell culture (Laski et al., 1984), yeast cells (Edwards & Schimmel, 1990), *Caenorhabditis elegans* (Li et al., 1998; Pilgrim & Bell, 1993), plants (Z. Chen et al., 1998), rabbit tissue culture (Chittum et al., 1998; Hatfield et al., 1988), the silkworm *Bombyx mori* (Srinivasan & Gopinathan, 2001), and mouse heart (Buvoli et al., 2000). It was also found that some viruses use stop codon readthrough to increase their limited genetic information by coding for multiple protein products with one transcript (Beier & Grimm, 2001; Cremer et al., 1979; Gesteland et al., 1977; Pelham, 1978; Philipson et al., 1978; Rawlins et al., 1983; Tuite et al., 1981; Yoshinaka et al., 1985). In parallels to CRISPR/Cas9 technologies, the extension of sup‐tRNAs to cultured human cells gave the first indication that their activity could be harnessed for the treatment of human diseases resulting from nonsense mutations (Temple et al., 1982). Nonsense codons were also used in conjunction with sup‐tRNAs to incorporate amino acids with novel side‐chain chemistries into proteins (Furter, 1998; Noren et al., 1989; L. Wang, Brock, et al., 2001). Much of the work on sup‐tRNAs in the past two decades has been focused on developing and improving methods to incorporate these novel amino acids.

## 5 USE OF NONSENSE SUP‐TRNAS FOR GENETIC CODE EXPANSION

Genetic code expansion (GCE) is characterized by the codon specific incorporation of noncanonical amino acids (ncAAs) into proteins in cells and animals. These ncAAs contain side chains that are chemically distinct from canonical amino acids, which provide additional chemical functionalities including reactive handles for further modification, spectral or imaging probes, photo‐ or chemical‐induced crosslinkers, or natural post‐translational modifications or structural mimics thereof (Dumas et al., 2015). GCE requires a “blank” codon that can be used as an incorporation signal for the ncAA. The most commonly used “blank” codon for GCE is traditionally the amber stop codon (UAG) because it is the least frequently used termination codon in the *E. coli* genome, although the other stop codons (Dumas et al., 2015), quadruplet codons (J. C. Anderson et al., 2004; Neumann et al., 2010), in some cases sense codons (K. Y. Fang et al., 2018; Schmitt et al., 2018), and even codon–anticodon pairs with noncanonical nucleotides (Feldman et al., 2019) have been employed for GCE.

In using a GCE system, the ncAA is introduced to the cell where an engineered aminoacyl‐tRNA synthetase/transfer RNA (aaRS/tRNA) pair that is orthogonal (i.e., does not cross‐react) to all intracellular aaRS/tRNA is employed to charge the orthogonal tRNA (o‐tRNA) with the ncAA (Figure 1(a)). The charged o‐tRNA is transported to and decoded by the ribosome in response to a blank codon encoded in the messenger RNA (mRNA) of a gene of interest. This leads to the co‐translational installation of an ncAA at a genetically defined position in the protein (Figure 1(b)). The use of sup‐tRNAs as nonsense therapeutics, much like their use for GCE, takes advantage of the amino acid and site‐specificity of the translational apparatus for the seamless rescue of PTCs. The development of the GCE field over the last 20 years has illuminated both the capabilities and many of the inefficiencies associated with the function of sup‐tRNAs in cells and animals.

## 6 CAPABILITIES AND INEFFICIENCIES ASSOCIATED WITH THE USE OF SUP‐TRNAS

Implementation of the majority of GCE systems requires the conversion of the o‐tRNA anticodon for nonsense suppression. Many aaRSs recognize the anticodon of their cognate tRNA as an identity element, and many of those that do not explicitly interact with the anticodon maintain extensive contacts with the tRNA body that are altered by changing the anticodon through long‐range structural perturbations (Hadd & Perona, 2014; Perona & Hadd, 2012). A variety of sequence substitutions were made to both the nonsense o‐tRNAs and their cognate o‐aaRSs to improve their interactions for increased efficiency of several different GCE systems in *E. coli*. These sequence modifications include mutations to the anticodon binding residues in the o‐aaRS and in the o‐tRNA anticodon stem (Ellefson et al., 2014; Guo et al., 2009; Hughes & Ellington, 2010; Rauch et al., 2016; Rogerson et al., 2015). These efforts have been undertaken because yields of ncAA containing protein are generally lower than that of WT protein. The exact yield as compared to WT protein production is often not quantified in the literature, however, for those that have been reported, they range from a small fraction of WT protein production (Park et al., 2011) to near WT levels (Schmied et al., 2014). Nonsense suppression efficiency for GCE in eukaryotes is influenced by many factors some of which include the promoter strength of the target gene of interest, o‐tRNA copy number and sequence, the efficiency of the evolved o‐aaRS, and the location of the PTC within the target gene of interest. As GCE systems are fully synthetic and all components must be introduced to the cell it is possible to tune these factors to increase the ncAA‐containing protein yield. When the pyrrolysine o‐tRNA/o‐aaRS was first implemented in human cell culture, the o‐aaRS and superfolder‐GFP‐TAG‐150 (sfGFP150TAG) were both expressed from CMV promoters with a 4‐copy CMVenhancer‐U6‐o‐tRNA. This initial o‐aaRS/o‐tRNA system gave a 5% yield of superfolder‐GFP as compared to WT (Schmied et al., 2014). A subsequent iteration of this o‐aaRS/o‐tRNA system was improved by expressing the o‐aaRS and sfGFP150TAG from EF1a promoters and including an 8‐copy U6‐o‐tRNA expression cassette. By altering the protein promoters and optimizing the o‐tRNA expression the yield of sfGFP150TAG was improved to 62% of WT (Schmied et al., 2014). This improvement highlights the importance of optimizing the ratios of the engineered translational components being delivered to the cell for optimal PTC suppression efficiency. While PTC sequence context and position within the gene transcript have been shown to influence PTC readthrough efficiency, no strict rules on rescued protein yields from genes containing PTCs have been determined. Some work has also been conducted to increase the function of o‐tRNA/o‐aaRS pairs in mammalian cells. For example, mutating the anticodon‐binding region of the *E. coli* TyrRS showed an ~2‐fold higher efficiency at charging the *E. coli* amber (UAG) sup‐tRNA^Tyr^ (tRNA^Tyr^_CUA_) as compared to the WT TyrRS in mammalian cells (Takimoto et al., 2009). Rational design of the *Methanosarcina mazei* pyrrolysine o‐tRNA in an effort to improve its interactions with the eukaryotic translational machinery resulted in a 2‐ to 5‐fold increase in ncAA incorporation in mammalian cells (Serfling et al., 2018). Even with the previously mentioned improvements, it has become clear, particularly in mammalian cells, that multiple copies of the nonsense suppressor o‐tRNA per copy of o‐aaRS are required for efficient function due to the inefficiencies associated with the interactions between nonsense suppressor o‐tRNAs and their cognate o‐aaRSs (Mukai et al., 2008; Sakamoto et al., 2002; Schmied et al., 2014; B. Shen et al., 2011; Zheng et al., 2017). As GCE in eukaryotic cells requires importing o‐tRNAs from phylogenetically distant organisms to maintain orthogonality and lack endogenous transcription elements (discussed below), alternate approaches for expression of o‐tRNAs are therefore necessary, with the main approach including the use of extragenic RNA polymerase III promoters U6 and H1 (Mukai et al., 2008; W. Wang et al., 2007).

With the steady development of GCE, first achieved in *E. coli* (Furter, 1998; L. Wang, Czaplinski, et al., 2001), its successful implementation has been realized in a number of cell types and organisms, including yeast (Hancock et al., 2010; Q. Wang & Wang, 2008), eukaryotic cell culture (Elsasser et al., 2016; Gautier et al., 2010; Hino et al., 2005; Mukai et al., 2008; Sakamoto et al., 2002; Xiao et al., 2013), *C. elegans* (Greiss & Chin, 2011; Parrish et al., 2012), Drosophila (Bianco et al., 2012; Elliott et al., 2014), zebrafish (Brown & Deiters, 2019; Chen et al., 2017; Liu et al., 2017), and mouse brain (Ernst et al., 2016; Kang et al., 2013; Maywood et al., 2018; Zheng et al., 2017). To implement GCE in these cells and organisms, the o‐tRNA was delivered in several different fashions including transient transfection (Hino et al., 2005; Sakamoto et al., 2002; Schmied et al., 2014; Xiao et al., 2013), viral transduction (Chatterjee et al., 2013; Shen et al., 2011; Zheng et al., 2017), genomic integration (Elsasser et al., 2016; Sakamoto et al., 2002), or injected as RNA (Infield et al., 2018; Noren et al., 1989; Saks et al., 1996). GCE methods have demonstrated that o‐tRNAs can be delivered with different methods as either DNA or RNA, and are stable, heritable, and tolerable in a variety of eukaryotic cells, zebrafish, and mice (Chen et al., 2017; Elsasser et al., 2016).

When the impact of expression of amber sup‐tRNA o‐tRNA expression in live transgenic mice was evaluated in tissues including the brain, heart, liver, colon, kidney, skeletal muscle, and lung, no morphological changes were noted. The liver transcriptomes showed minor changes with 97 upregulated and 47 downregulated transcripts. These genes were mostly related to metabolism but did not impair liver function (Chen et al., 2017). Given the minimal impact of GCE machinery in live animals, we expect that sup‐tRNAs for the incorporation of canonical amino acids will also be well tolerated.

While the development of GCE has outlined many of the capabilities of sup‐tRNAs as described above, it has also highlighted some of the transcriptional and translational inefficiencies associated with the use of sup‐tRNAs and genes containing nonsense mutations. Nonsense‐mediated decay (NMD) and no‐go decay (NGD) are eukaryotic translation‐dependent mRNA quality control pathways that selectively degrade mRNAs containing a PTC (Kurosaki et al., 2019; Popp & Maquat, 2013; Shoemaker & Green, 2012). Use of the amber stop codon for the incorporation of ncAAs subjects the mRNA to degradation, which generally decreases protein yield. An NMD‐deficient yeast strain made by knocking out UPF1, an essential NMD factor, increased ncAA incorporation by more than 2‐fold as compared to WT yeast (Q. Wang & Wang, 2008). Similarly, in *C. elegans*, inhibition of NMD led to stabilization of amber‐stop‐codon‐containing mRNA and increased ncAA‐containing protein expression (Greiss & Chin, 2011; Parrish et al., 2012). Furthermore, sup‐tRNAs need to compete with the endogenous translational termination machinery. Translation termination in eukaryotes normally occurs in response to an NTC in the ribosomal A‐site requiring two release factors (RFs) eRF1 and eRF3 in complex with GTP. The eRF1/eRF3/GTP complex interacts with the ribosome via eRF1 that is responsible for codon recognition. Following GTP hydrolysis by eRF3, eRF1 hydrolyzes the polypeptidyl‐tRNA, releasing the completed protein product (Jackson et al., 2012). This process also occurs at PTCs and leads to the release of incomplete polypeptides from the ribosome, often resulting in a loss‐of‐function of the protein. A dominant‐negative mutation of eRF1 was developed as a method to increase the yield of full‐length ncAA‐containing protein in eukaryotes, with the eRF1 E55D mutation increasing yields up to 5‐fold (Schmied et al., 2014). It has also become clear from the GCE field that the sequence context of PTCs and NTCs in mRNA is important for determining the level of suppression (Chemla et al., 2018; Johnson et al., 2011; Schwark et al., 2018; Xu et al., 2016). While much of this work has been conducted in *E. coli* and the exact determinants underlying efficiency of stop codon suppression are likely different between *E. coli* and eukaryotes, the importance of stop codon context, in general, is transferable (Lee & Dougherty, 2012; Wangen & Green, 2020).

## 7 THE IMPACT OF MAMMALIAN TRNA BIOGENESIS ON THE APPLICATION OF SUP‐TRNAS

**FIGURE 2:**
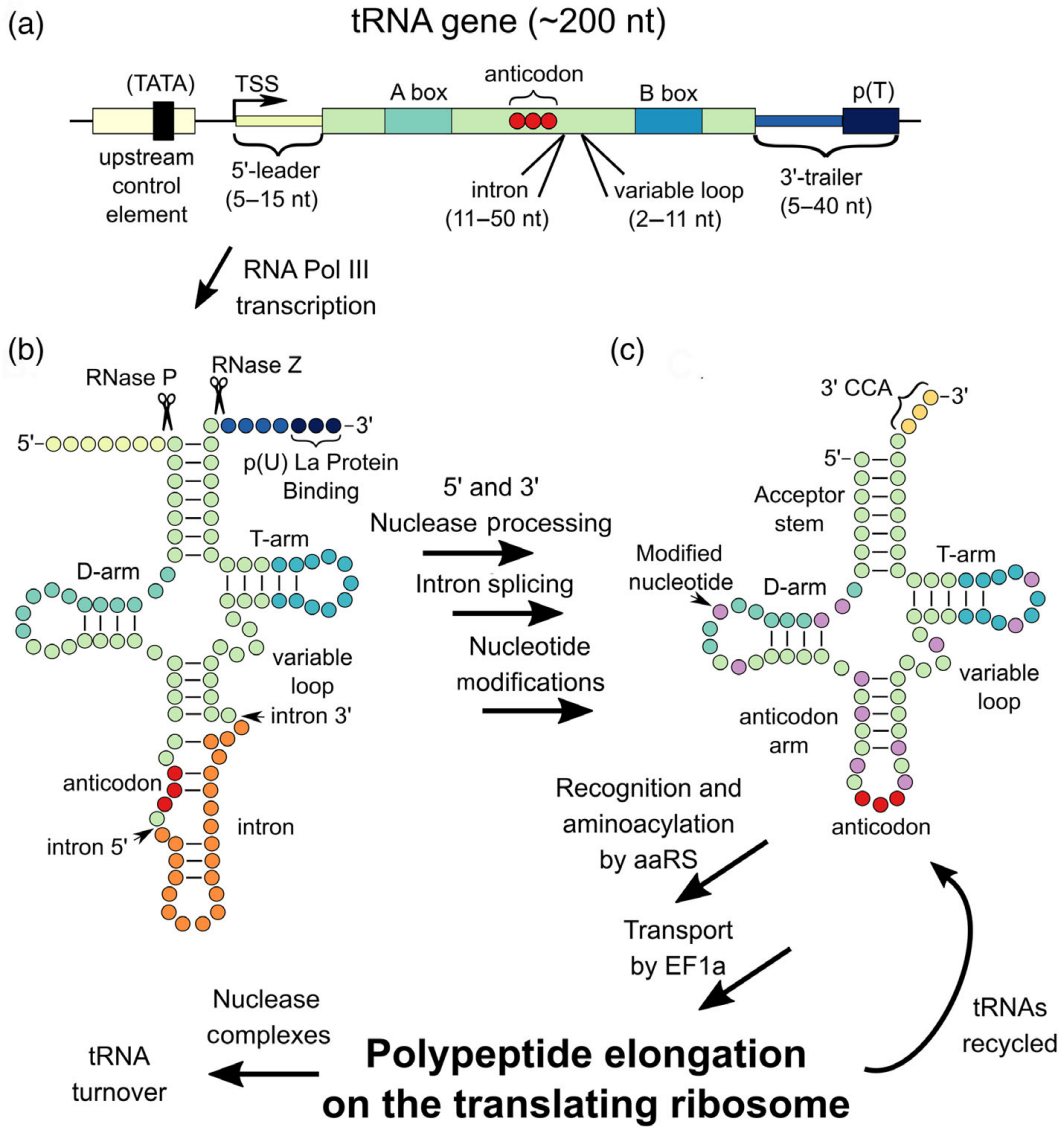
Diagram of structural and functional elements of a mammalian tRNA gene, processes involved with mammalian tRNA biogenesis, and tRNA function in translation. (a) Diagram of a typical tRNA gene containing an upstream control element containing a TATA‐box, transcription start site (TSS), 5′‐leader sequence, tRNA sequence containing internal A‐ and B‐box promoter elements, the location of the anticodon, intron, and variable loop, 3′‐trailer, and poly‐thymidine transcriptional terminator (p[T]). (b) Mammalian tRNAs are synthesized as a primary RNA transcript containing 5′‐leaders removed by RNase P, introns spliced out by the SEN complex, and 3′‐trailer removed by RNase Z or other exonucleases. The RNA chaperone La protein binds the poly‐uridine tracts at the 3′ ends of the primary transcript to stabilize the transcript and direct processing. The 3′‐terminal CCA trinucleotide common to all tRNAs is ligated to the pre‐tRNA during processing. Throughout the tRNA maturation process, 10–15 nucleotides are chemically modified by specific modification enzymes. (c) Following processing, tRNAs are aminoacylated by their cognate aminoacyl‐tRNA synthetase (aaRS), transported to the ribosome by EF1a, and finally participate in translation on the ribosome

Understanding how tRNAs are processed in mammalian cells is crucial to fully harness the potential of sup‐tRNAs and for their engineering to be ultimately used as therapeutic. Due to the ease of genetic manipulations in yeast, the majority of our current understanding of eukaryotic tRNA biogenesis stems from studies in yeast. When considering sup‐tRNAs for nonsense therapeutics it is important to note the differences in tRNA biogenesis between yeast and mammalian cells. RNA polymerase III (Pol III) transcribes all known eukaryotic nuclear tRNA genes. For transcription of tRNA, the Pol III machinery makes use of promoter elements located within the transcribed region. These type II internal RNA Pol III promoters are composed of an A‐box and B‐box (Figure 2(a); Dieci et al., 2007). While the A‐ and B‐boxes alone are sufficient for transcription, an upstream control element generally containing a degenerate TATA‐box in mammals also modulates RNA Pol III transcription in a cell‐type and developmentally controlled manner (Dittmar et al., 2006; Giuliodori et al., 2003; Zhang et al., 2011). Pol III terminates at short runs of T residues (T ≥ 4 in vertebrates) in the absence of accessory factors (Richard & Manley, 2009). Termination at such signals generates a variable‐length 3′ oligo(U) tract on Pol III transcripts like tRNA. A tRNA chaperone, the oligo(U)‐binding protein La, helps to stabilize the nascent transcript and direct processing (Maraia & Lamichhane, 2011).

These primary RNA transcripts or pre‐tRNAs, contain 5′‐leaders and 3′‐trailers of variable length that must be removed during tRNA maturation before they participate in translation (Figure 2(b)). The pre‐tRNA 5′‐leader is removed by the endonuclease RNase P (Walker & Engelke, 2006). The major pathway for tRNA 3′ end processing is the removal of 3′‐trailer by the endonuclease RNase Z, followed by addition of the CCA trinucleotide after terminal nucleotide 73 (N_73_; Vogel et al., 2005). In the absence of precise cleavage, several 3′ exonucleases can processively digest the 3′‐trailer up to N_73_ (Copela et al., 2008).

While tRNAs are generally similar in overall structure and are tuned for similar ribosomal binding (Ledoux & Uhlenbeck, 2008; Olejniczak et al., 2005), each pre‐tRNA contains the sequence information to direct the addition of a unique set of 10–15 specific post‐transcriptional modifications catalyzed by a wide variety of modification enzymes (Figure 2(c); Phizicky & Hopper, 2010). These tRNA modification enzymes need to recognize the sequence and/or structure of specific elements in a subset of tRNAs within the pool of cellular tRNA substrates. Properties that allow for tRNA modification enzymes to discriminate among tRNAs include pre‐existing modifications, presence of an intron, removal of an intron, and subcellular localization (Hopper & Phizicky, 2003; Kessler et al., 2018; Phizicky & Hopper, 2010). In humans, 6% of all tRNAs contain introns invariably found between nucleotides 37 and 38 (Lowe & Eddy, 1997; Phizicky & Hopper, 2010). The conserved splicing endonuclease (SEN) complex specifically splices out tRNA introns before tRNAs can participate in translation (Hirata, 2019). Post‐transcriptional modifications of tRNAs are important both for function in translation and stability (Hopper et al., 2010; Phizicky & Hopper, 2010).

Early tRNA processing steps occur in the nucleus and later steps occur after export of the tRNA to the cytoplasm by the tRNA‐specific factor Exportin‐t (Xpo‐t) or other less well‐characterized tRNA nuclear export factors (K. Chatterjee et al., 2017; K. Chatterjee et al., 2018; Kohler & Hurt, 2007). Due to the addition of order‐specific tRNA modifications, tRNAs are sometimes imported back into the nucleus in a process known as retrograde transport (Ohira & Suzuki, 2011; Shaheen et al., 2007). Re‐import of tRNAs back into the nucleus is also part of the tRNA quality control process prior to maturation (Hopper & Huang, 2015).

Mature tRNAs localized in cytoplasm are then recognized by their cognate aaRS and aminoacylated with their cognate amino acid. The specificity of this interaction, which is critical for maintaining translational fidelity, is mostly dependent on interactions between the aaRS and one or more of the discriminator base (N_73_), the acceptor stem, and the anticodon (Ibba & Soll, 2000). Interactions with other unique structural elements of certain tRNAs including wobble pairs, the variable loop, and specific nucleotide modifications are important in some cases (Cropp & Schultz, 2004). The aminoacylated tRNA is then bound by elongation factor 1 alpha (EF1a) with low nanomolar affinity (Gromadski et al., 2007) and transported to the A‐site of the translating ribosome to participate in peptide elongation (Negrutskii & El'skaya, 1998). Generally, tRNAs are stable macromolecules with the ability to participate in many rounds of translation as outlined above. However, if there are defects in the tRNA maturation or if damage occurs to the tRNA, there are several tRNA turnover pathways.

Throughout the tRNA biogenesis process, a variety of defects, including transcription errors, missing base modifications, or structural defects, can occur in tRNA transcription and processing. Aberrant tRNAs are recognized by the nuclear exosome complex, which flags defective tRNAs with a 3′ poly(A) tail (Kadaba et al., 2004), and are degraded by the nuclear exosome as part of a nuclear surveillance system (Schneider et al., 2007; Vanacova et al., 2005). Often, tRNA hypomodification results in structural defects, which are targeted for degradation by this surveillance system (Hopper et al., 2010).

These described tRNA biogenesis processes may be important when thinking about delivery of sup‐tRNAs as a therapeutic. To deliver sup‐tRNAs as DNA therapeutics, the expression cassettes should contain sequences that promote proper upstream control elements for efficient transcription in the cell type of interest, 5′‐ and 3′‐processing, and post‐transcriptional processing and modifications. If delivered as a DNA, whether by virus or naked DNA, the therapeutic must first be trafficked to the nucleus for transcription. When delivered as RNA, it is still unknown if simply delivering to the cytoplasm is sufficient for proper modification. Will it be necessary to deliver sup‐tRNAs as RNA or encoded as DNA to the target cell's nucleus to be a robust nonsense therapeutic? How do modifications influence the stability and function of sup‐tRNAs? It is likely that therapeutic delivery routes will need to support both efficient cellular delivery and proper downstream sup‐tRNA processing and modification.

## 8 KEY ASPECTS OF TRNA PROCESSING AND INTERACTIONS IN THE TRANSLATION MACHINERY FOR OPTIMAL FUNCTION OF SUP‐TRNAS IN VIVO

For sup‐tRNAs to function in vivo as a therapeutic, they will need to interact favorably with the transcription and/or translation apparatus in the cell. When delivered as DNA, the sup‐tRNA needs to be transcribed efficiently in the tissue of interest to treat a particular disease. As the upstream control element modulates RNA Pol III transcription in a cell‐type dependent manner, selection of the proper regulatory element is necessary to give optimal expression in the cell type(s) of interest. Following transcription, the pre‐tRNA needs to be efficiently processed at the 5′ end by RNase P, the 3′ end by RNase Z or other exonucleases, and therefore need to contain the necessary elements. As introns are important for direction of other modifications, their inclusion or exclusion in the pre‐tRNA sequence should also be considered for efficient processing.

Whether delivered as DNA or RNA, post‐transcriptional nucleotide modifications are important for maintaining tRNA therapeutic stability and optimal interactions with the translation apparatus. As previous processing steps like intron splicing and nucleotide modifications are important for catalysis of downstream modifications, it will be important to determine the hierarchy of these modifications in the context of sup‐tRNAs. This may be important for stability of the sup‐tRNAs as hypo‐modifications may result in increased tRNA degradation and reduced intracellular steady‐state sup‐tRNA levels (Hopper et al., 2010).

Proper interactions between the sup‐tRNA with the cognate aaRS are critical for maintaining translational fidelity and nonsense codon suppression efficiency of the sup‐tRNA therapeutic. To produce a functional sup‐tRNA, the anticodon needs to be altered such that it suppresses nonsense codons in mRNA on the ribosome. As the anticodon is often a major determinant of aaRS recognition, it is important to have a strategy to determine sup‐tRNA sequences that maintain optimal interactions between a tRNA and its cognate aaRS and disfavor interactions with any off‐target non‐cognate aaRSs.

In our previous work, we approached this by identifying every codon that is a single nucleotide different from one of the three stop codons. We then determined human tRNA isoacceptors (all tRNA sequences, whether differences in the anticodon or tRNA body, for a particular amino acid) within these codon families and converted all of the anticodons to suppress nonsense codons, which we called anticodon‐edited tRNAs (ACE‐tRNAs, Figure 2(a) and (c)). Using an all‐in‐one high throughput cloning and screening vector containing a PTC‐interrupted NanoLuciferase, we screened these ACE‐tRNAs in HEK293 cells to determine which sup‐tRNA sequences supported the highest PTC suppression activity (Lueck et al., 2019). Therefore, this screening method determined which ACE‐tRNAs were expressed and/or interacted most favorably with all of the translation components, including the aaRS, despite alterations made to the anticodon. To ensure our ACE‐tRNAs maintain translational fidelity and seamlessly suppress PTCs, that is insert the correct amino acid, we performed mass spectrometry on the rescued full‐length protein. For our top hits from the TrpTGA and GlyTGA ACE‐tRNA families, we confirmed high‐fidelity incorporation of their cognate amino acids at the PTC site, indicating preservation of translational fidelity of our ACE‐tRNAs (Lueck et al., 2019). This indicates that the ACE‐tRNAs as a therapeutic will seamlessly rescue WT protein function by reverting the PTC to the original amino acid coded for. Western blot analysis of HEK cells co‐transfected with a plasmid containing four copies of the best‐performing ACE‐tRNA_Gly_ and pcDNA3.1(+) G542X‐CFTR showed that 20% of full‐length CFTR expression was rescued as compared to WT CFTR, while a similar experiment with W1282X‐CFTR and ACE‐tRNA_Trp_ revealed a full‐length protein rescue of 6%. Further experiments co‐injecting *Xenopus* oocytes with CFTR mRNA and ACE‐tRNA (as RNA) displayed 100% rescue of WT CFTR function for G542X CFTR mRNA with injection of 100 ng of ACE‐tRNA_Gly_. As an example of the variability of sup‐tRNA activity, in the same study, only 50% of W1282X‐CFTR function was rescued following injection of 100 ng ACE‐tRNA_Trp_ as RNA (Lueck et al., 2019). Indeed, the general activity of sup‐tRNAs is difficult to define, because it is dependent on the sup‐tRNA amino acid family, its delivery modality, and target PTC. Indeed, there is an open question about the ability of sup‐tRNAs to promote enough readthrough of disease‐causing PTCs to surpass a therapeutic threshold.

While our screen was a good starting point for identifying functional sup‐tRNAs, there are several facets of tRNA biogenesis that may influence their therapeutic promise. First, the screen was performed in HEK293 cells. It has been shown that tRNA transcription is influenced by tissue type (Torres et al., 2019), and therefore, different therapeutic indications may necessitate different sup‐tRNAs sequences that are optimal for the target cells. Furthermore, the screen was focused on sup‐tRNA potency of PTC suppression within 24–48 h but not persistence of activity. Additionally, the screen was performed by cDNA transfection, where delivery method may have differential impact on sup‐tRNA expression, processing, and downstream PTC suppression activity. Currently, we do not have a firm grasp on which aspects of tRNA biogenesis need to be considered for development of a PTC therapeutic and which of those can be ignored.

Most codons code for the addition of an amino acid to the growing polypeptide chain, while NTCs signal for this process to stop. NTCs signal to stop by binding to release factors, which cause the ribosome to dissociate, releasing the polypeptide chain. Due to the presence of release factors in the cell, sup‐tRNAs are in competition with release factors for suppression of PTCs versus translation termination. The translation termination machinery has been targeted with antisense oligos as a potential treatment option for nonsense‐associated diseases (Huang et al., 2019). While downregulation of the translation termination or NMD machinery in conjunction with sup‐tRNA delivery will inhibit the degradation of aberrant mRNAs and termination at NTCs, which may negatively affect cell health, given that all three approaches are generally well tolerated, it is possible that strategies targeting the translation termination or NMD machinery could be used in combination with sup‐tRNAs to bolster rescue of full‐length protein expression.

## 9 POTENTIAL COMPLICATIONS DUE TO OTHER ROLES OF TRNA IN MAMMALIAN BIOLOGY

Although tRNAs are best known for their role in translation, the whole picture of the tRNA world seems much more complex than anticipated. Recent reviews hypothesize additional regulatory tRNA functions in a variety of cellular processes beyond protein biosynthesis, for example, gene expression, protein degradation, and apoptosis (Kirchner & Ignatova, 2015; Raina & Ibba, 2014; Schimmel, 2018). Most of these processes are stress‐induced and in light of sup‐tRNA overexpression, these additional functions might pose possible risks. Aminoacylated tRNAs are not only substrates for ribosomal translation but also for post‐translational protein labeling and degradation. tRNA‐dependent addition of a primary destabilizing amino acid to the N‐terminus of proteins makes them a target for the cellular degradation machinery. Sup‐tRNAs are appropriate substrates, since the aminoacyl‐transfer RNA–protein transferases only interact with the aminoacyl moiety for recognition, thus high sup‐tRNA levels in the cell may trigger increased protein degradation and cellular stress. In a stress response, uncharged tRNAs can function as regulators of protein synthesis and global gene expression levels in the cell. Small noncoding RNAs play also an important role in regulatory cellular processes. Although tRNAs are among the most stable RNA molecules in the cell, they can give rise to small tRNA fragments in the tRNA maturation process. Growing evidence suggests that these tRNA derived small RNA fragments are involved in cell regulation processes like protein synthesis, gene expression, and apoptosis as well. Furthermore, increasing the expression of low‐abundance tRNAs can alter the folding and solubility of many cellular proteins, which may decrease cell fitness (Kirchner & Ignatova, 2015) and overexpression of tRNA is observed in cancer cells (Raina & Ibba, 2014).

## 10 LIMITATIONS OF SUP‐TRNAS TO BE USED IN GENE THERAPY

Despite successful reports of nonsense suppression using sup‐tRNAs in vivo, this approach has not yet reached the translational stage. To use sup‐tRNA‐based PTC suppression as a gene therapy, the delivery hurdle must be overcome, and a therapeutic window must be defined with possible toxic side‐effects identified. In the following chapters, we are going to highlight and address some of the major concerns raised in the application of sup‐tRNAs as therapeutics.

## 11 DESIRABLE QUALITIES OF A SUP‐TRNA AS A THERAPEUTIC

As discussed, suppression efficiency of sup‐tRNAs is influenced by several factors: (i) competition with release factor complex, (ii) efficiency of aminoacylation by endogenous aminoacyl‐tRNA synthetases (aaRSs), (iii) binding affinity to eEF1A, (iv) half‐life of sup‐tRNA, (v) influence of PTC sequence context effects, and (vi) steady‐state mRNA expression levels of the target gene.

To overcome many of the sup‐tRNA inefficiencies, increasing tRNA expression has been the most heavily employed strategy traditionally. Several studies, including GCE, have shown that increasing the level of sup‐tRNAs in the cell improves nonsense suppression levels. Increasing expression of sup‐tRNAs leads to a larger intracellular pool of aminoacylated sup‐tRNAs, which results in greater competition with eRF1 for suppression of PTCs on the translating ribosome. Sup‐tRNAs have been delivered as plasmids containing anywhere from 4 to 16 copies of the sup‐tRNA expression cassettes per plasmid into patient‐derived cells as well as mouse skeletal muscle and heart (Buvoli et al., 2000; Panchal et al., 1999), which indeed results in higher PTC suppression efficiency. However, overexpression of sup‐tRNAs may have toxic effects on cellular metabolism and therefore therapeutic application may require stringent control over the sup‐tRNA level (Kiselev et al., 2002). In contrast, and in support of in vivo sup‐tRNA use as a therapeutic, it has been shown that high levels of amber (UAG) sup‐tRNA expression in transgenic mice did not seem to have an overt impact on tissue morphology and was well tolerated in GCE studies. Clearly, the expression levels of sup‐tRNAs is important for PTC suppression efficiency, and therefore is inextricably linked to the mode of delivery and efficiency.

## 12 NONSENSE‐MEDIATED DECAY DETERMINES THE NUMBER OF TRANSCRIPTS AVAILABLE FOR RESCUE BY SUP‐TRNAS

There is an important distinction between therapeutic approaches for missense and nonsense diseases. With missense mutations, the therapeutic target is predominately an aberrantly functioning protein. With nonsense mutations, there is a complete loss of protein, and therefore the target is shifted towards mRNA. The rescue of target gene protein expression by sup‐tRNAs necessitates sufficient expression of target gene mRNA. As previously discussed, NMD is a highly conserved pathway for quality control of transcripts in eukaryotic cells (Kurosaki et al., 2019). It involves the identification and degradation of faulty transcripts and leads to very low levels of PTC‐bearing mRNAs. For example, cystic fibrosis causing nonsense mutations can reduce the steady‐state levels of CFTR transcripts to 10%–20% of WT. The impact of NMD on transcript expression is the focus of ongoing PTC therapeutic studies. The NMD inhibitor Amlexanox is already approved by the FDA (Atanasova et al., 2017; Gonzalez‐Hilarion et al., 2012; Makino et al., 1987). It has been shown that even partial NMD inhibition significantly increases the steady‐state transcript levels (Bedwell et al., 1997; Bellais et al., 2010; Floquet et al., 2012; Linde et al., 2007; Salvatori et al., 2009). Furthermore, combinatorial therapies of aminoglycosides with NMD inhibitors increased expression of protein (Durand et al., 2007; Gotham et al., 2016; Keeling et al., 2013; Linde et al., 2007; Usuki et al., 2004; Usuki et al., 2006; W. Wang, Czaplinski, et al., 2001). Altering NMD levels must be approached with caution as NMD is involved in transcriptome regulation and embryonic development (Y. Fang et al., 2013; Huang & Wilkinson, 2012; Hwang & Maquat, 2011; Nguyen et al., 2014). In studies in mice, compromised NMD results in embryonic lethality (McIlwain et al., 2010; Medghalchi et al., 2001) and intellectual disability (Nguyen et al., 2013; Tarpey et al., 2007), whereas partial inhibition of NMD after embryonic development showed no detrimental effects (Frischmeyer‐Guerrerio et al., 2011; Usuki et al., 2013).

## 13 THE DELIVERY HURDLE

**FIGURE 3:**
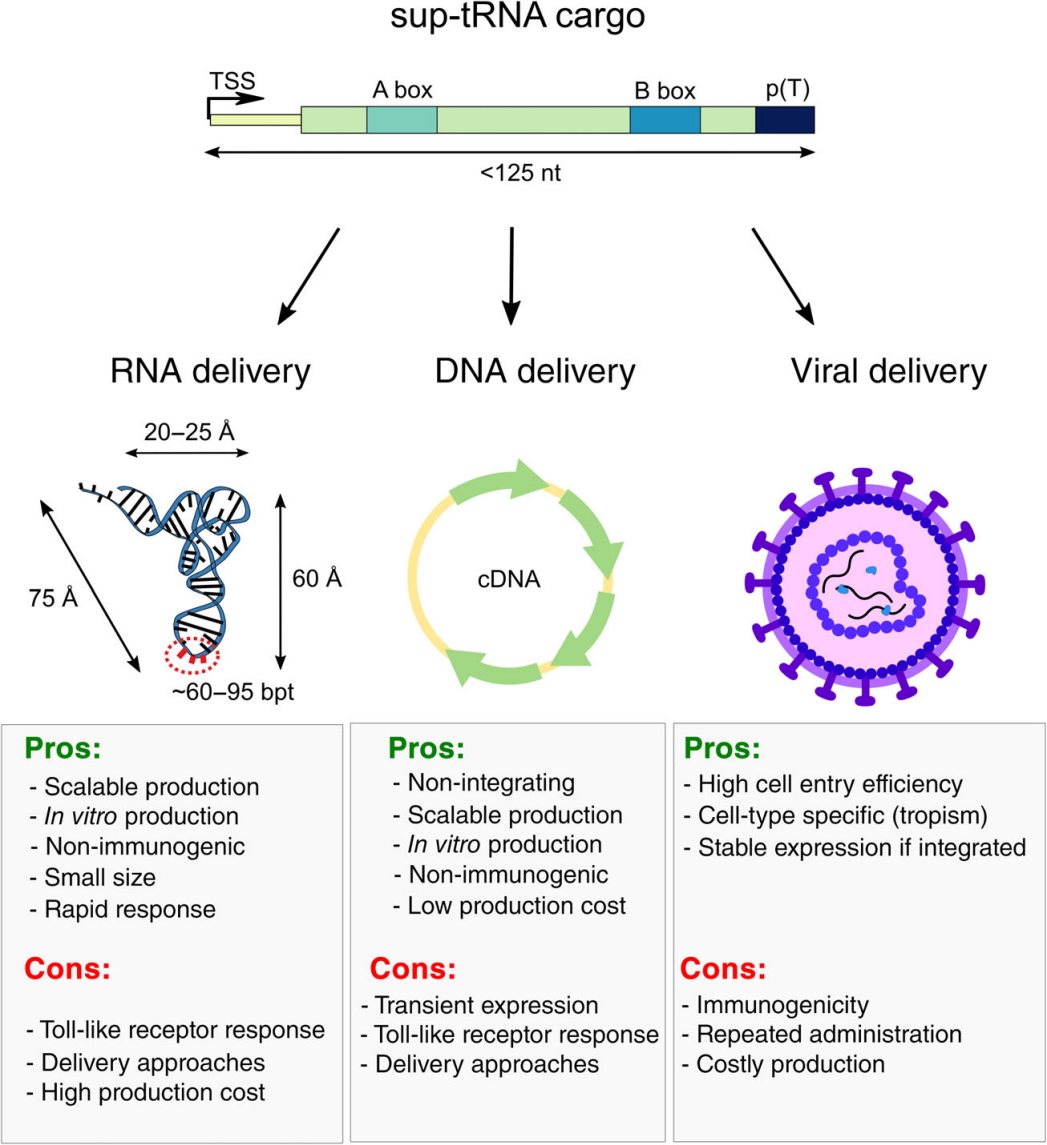
Pros and cons of sup‐tRNA delivery options. For specific elements necessary for sup‐tRNA expression from DNA or virus see Figure 2(a) or Lueck et al. (2019)

As with every therapeutic application, finding an appropriate delivery strategy is a crucial step towards its success. The compact gene structure of sup‐tRNAs allows for the use of multiple different delivery modalities including DNA or RNA, each of which affords several delivery options (Figure 3). The therapeutic needs to be delivered to the target cells and permeate the cell membrane to reach its destination of action. The particular delivery strategy also has an impact on the kinetic and dynamic profile of the therapeutic. Sup‐tRNA therapeutics can be divided into non‐viral and viral delivery as well as DNA‐ or RNA‐based delivery.

### 13.1 Viral delivery of sup‐tRNA

There is a broad spectrum of viral vectors that include adenovirus, adeno‐associated virus (AAV), herpes simplex, retrovirus, lentivirus, alphavirus, flavivirus, rhabdovirus, measles virus, Newcastle disease virus, poxvirus, and picornavirus, all of which have different strengths for use as viral gene therapy agents (Lundstrom, 2018). Each one of these viruses has features including tropism, cargo capacity, ability to integrate, and immunogenicity that make them well suited for specific diseases. Viral delivery is commonly used in gene therapy as viral DNA cargo delivery is usually much more efficient than non‐viral delivery and has the advantage of cell‐type‐specific transduction. Newly developed viral delivery strategies targeting specific cell types have lessened the general safety concerns associated with the use of viral vectors in humans (Lehrman, 1999). For the purpose of this review, we will not do a deep dive on viral therapy approaches as there are exceptional reviews elsewhere (Goswami et al., 2019; Lundstrom, 2018; van Haasteren et al., 2020). Revisiting the idea that total gene replacement is the “gold standard” of gene therapy, a significant limitation is that often the gene to be replaced exceeds the cargo capacity (i.e. payload) of the virus. For example, AAV has cargo capacity of <4.7 kb and has resulted in FDA approved therapies for nonsense associated disease LCA where the *rpe65* gene at 1.5 kb coding sequence (CDS) is well within the cargo capacity. Whereas for diseases such as Stargardt's disease, Usher syndrome, and Duchenne muscular dystrophy the CDS exceeds the cargo capacity of AAV and many other large capacity viruses. In addition, the use of sup‐tRNAs retains all of the genomic regulatory components for the PTC‐containing genes, lessening the potential for cellular dysregulation following overexpression of delivered CDS under control of exogenous promoters. This highlights the perceived strengths of the diminutive size of sup‐tRNA expression cassettes. To implement sup‐tRNA approaches, the minimum expression cassette is approximately 140 bp including the tRNA leader, sup‐tRNA, and termination sequence, allowing for up to 35 expression cassettes per AAV vector. Barring DNA instability and rearrangement, viral cargo capacity is not a limitation for sup‐tRNAs and therefore can be used to target disease‐causing PTCs in genes of any size. This provides the opportunity to pair the best viral vector approach for each disease and tissue type without concern for viral cargo capacity.

### 13.2 Delivery of sup‐tRNA as DNA

Naked DNA delivery is an attractive gene therapy approach as it is inherently low immunogenicity, which allows for re‐dosing, can be designed to remain episomal or integrate, has no size constraint, and can be paired with several delivery technologies that include nanoparticle formulations, liposomes, pressure injection, and electroporation. As with viruses there are several excellent reviews with a detailed discussion of this topic (Dean, 2005; Ramamoorth & Narvekar, 2015; Shen et al., 2019). Perhaps the best‐developed use of naked DNA in gene therapy is its use in DNA vaccines. One of the more developed delivery methods is electroporation. At present, 222 clinical trials are registered using electroporation in humans. These include 94 trials for gene therapy mostly in muscle, skin, and tumors, two of which are in phase III. Naked DNA delivery does not come without issues as it can be silenced due to methylation, diluted from cell division, and induce a myriad of cell responses including Toll‐like receptor response (Dean, 2005). It should also be noted that cytoplasmic delivery of DNA to target cells is not sufficient, as therapeutic DNA must enter the nucleus for transcription. Therefore this requirement must be considered when developing naked DNA delivery strategies (Dean, 2003). Non‐viral delivery has the advantage of cost‐effective scalable in vitro production of large amounts of synthetic DNA for transfection. It is considered a safe method for gene transfer, because unlike viral delivery, nucleic acids transferred to the cell pose less risk of insertional mutagenesis. A perceived limitation of naked DNA therapeutics is a relatively short‐expression half‐life. However, several studies have shown extended‐expression (months) in murine lung following plasmid DNA (pDNA) delivery by polyethylimine (PEI)/DNA complexes (Bazzani et al., 2016) pDNA/cationic liposome complexes (Gill et al., 2001), and electroporation (Dean, 2005), when pDNA sequences are made resistant to silencing. Since non‐viral delivery of nucleic acids shows less immunogenicity than viral delivery, repeated administration can be performed. In principle, the size of the transgene is unlimited, but intracellular exogenous DNA mobility and nuclear entry is correlated negatively with plasmid size (Hornstein et al., 2016; Lukacs et al., 2000; Mieruszynski et al., 2015). Arguably, the simplest method of gene delivery is injection of naked DNA, which has been used to successfully transduce liver (Herweijer & Wolff, 2003). Sup‐tRNAs encoded and delivered as naked DNA provides the opportunity for optimization of sup‐tRNA expression cassette copy number and vector size to facilitate efficient cell entry, intracellular mobility, and high levels of sup‐tRNA expression. These sup‐tRNAs can then be paired with emerging naked DNA delivery technologies.

### 13.3 Delivery of sup‐tRNA as RNA

Sup‐tRNA technology allows for delivery as RNA, which is its active form. As mentioned above, tRNAs are transcribed from DNA in the cell nucleus and post‐transcriptionally processed and modified. Post‐transcriptional modifications are crucial for stability, or sup‐tRNA half‐life, and likely important for PTC suppression efficiency. However, it has been shown that cytoplasmic delivery of unmodified sup‐tRNA supports robust PTC suppression (Infield et al., 2018; Lueck et al., 2019), and therefore unmodified sup‐tRNA could be considered a pro‐drug, that is further modified to its highly active form once it has entered the target cell (see above). However, as with DNA, delivery of RNA can also elicit a Toll‐like receptor response (Dalpke & Helm, 2012). Therefore, most delivery of RNA requires extensive nucleotide backbone modifications to escape this undesired cellular response, which may also be a necessary step for delivery of sup‐tRNAs as RNA (Freund et al., 2019). Delivery of sup‐tRNA using viral and DNA vectors supports transcriptional amplification of cellular sup‐tRNA levels. However, when sup‐tRNAs are delivered as RNA, the delivery efficiency dictates cellular sup‐tRNA levels. This does not mean that sup‐tRNAs delivered as RNA are not a renewable PTC suppression resource. Indeed, each sup‐tRNA can go through multiple rounds of aminoacylation and translation. However, the persistence as a therapeutic may be dramatically shortened when delivered as RNA, as it is inextricably linked to sup‐tRNA half‐life. Therefore, it is likely that repeated delivery of sup‐tRNA RNA would be needed to sustain a therapeutic level of target protein expression. Because sup‐tRNAs are only ~76 nucleotides in length, many of the existing mRNA and short RNA delivery technologies can be used. These include but are not limited to cationic or ionizable lipids, and lipid‐like materials, polymers/nanoparticles, cell‐penetrating peptides, and various PAMAM chemistries (Kowalski et al., 2019). The current status of these RNA delivery technologies can be found in several excellent reviews (Kowalski et al., 2019; Shin et al., 2018). Clearly, the choice of sup‐tRNA RNA delivery technology will depend on the target tissue of the clinical indication.

In principle, all described delivery strategies are feasible for sup‐tRNA therapeutics. Table 1 lists the strategies chosen in published sup‐tRNA studies summarized. The majority used cDNA injection for in vivo mouse models (Buvoli et al., 2000; Kiselev et al., 2002; Lueck et al., 2019), only two studies worked with sup‐tRNA as RNA, but only in cell‐based assays (Lueck et al., 2019; Sako et al., 2006). No data is available for application in large mammals (i.e., pigs), as sup‐tRNA studies have not reached this stage yet. It is hard to predict which might be the best delivery strategy for sup‐tRNAs. The 2017 FDA approval of LUXTURNA (*voretigene neparvovec‐rzyl*), a subretinally delivered AAV therapeutic for the treatment of the inherited retinal disorder Leber congenital amaurosis (LCA) type‐2, has shown the feasibility of viral‐mediated total gene replacement therapy for RPE65 (retinal pigment epithelial‐specific 65 kDa protein). Furthermore, it demonstrated a possible route to viral delivery of sup‐tRNAs for rescue of nonsense associated diseases of the eye where the target gene size exceeds the viral payload. The eye is an easily accessible target with constrained size. The blood‐retina barrier prevents widespread immune response, making it immune privileged. Since retinal cells are non‐dividing, non‐integrating virus vectors like AAV are sufficient for stable transgene expression. Treatment of airway epithelial cells in cystic fibrosis, for example, is another perceived low hanging fruit, due to its physical accessibility and constrained size. In animal studies, DNA has been successfully delivered as aerosol through the respiratory tract with subsequent electroporation. Delivery strategies for diseases like Duchenne muscular dystrophy are more complicated to address for all gene therapy approaches, because target cells are widespread throughout the body.

**TABLE 1:** Delivery strategies used in sup‐tRNA studies

Study	Disease	Genetic/protein target	Model organism	Delivery	tRNA copies
Temple et al. (1982)	β‐Thalassemia	β‐Globin chains	*Xenopus laevis* oocyte (in vitro)	DNA microinjection	
Panchal et al. (1999)	Xeroderma pigmentosum	Xeroderma pigmentosum group A (XPA) gene	XPA patient‐derived cells (XP12ROSV; in vitro)	HSV viral delivery	~15
Buvoli et al. (2000		Chloramphenicol acetyltransferase (CAT)	transgenic mice (in vivo)	direct DNA injection into skeletal muscle, tongue, and heart	8 or 16
			COS 7 cells (in vitro)	DNA transfection	
Kiselev et al. (2002)	Duchenne muscular dystrophy (DMD)	Dystrophin gene	C57B16J *mdx* mice (in vivo)	DNA + human lactoferrin carrier injection	
			HeLa cells (in vitro)	DNA polyplex (VSST‐525)	
Sako et al. (2006)	Ullrich disease	Collagen VI α2 gene	Patient‐derived cells (in vitro)	tRNA transfection	
Bordeira‐Carrico et al. (2014)	Hereditary diffuse gastric cancer (HDGC)	*CDH1/*E‐cadherin	AGS, MDA‐MB‐231, CHO cells (in vitro)	DNA transfection	1 or 5
Lueck et al. (2019)	Cystic fibrosis (CF)	Cystic fibrosis transmembrane conductance regulator (CFTR)	Mouse skeletal muscle (in vivo)	DNA injection + electroporation	4
			HEK293 cells (in vitro)	tRNA transfection	
			*Xenopus laevis* oocyte (in vitro)	tRNA microinjection	

Therapeutic threshold, the minimal amount of full‐length functional protein needed to gain a therapeutic effect, is different for each disease. Thus, the therapeutic threshold needs to be assessed for each gene and disease separately. Furthermore, the therapeutic threshold does not only differ between different diseases but can also significantly differ in the same disease between different tissues in one individual as well as between the same tissues in different individuals. Thus, a case‐to‐case analysis of nonsense suppression might be necessary. Some examples of therapeutic threshold: 30%–35% in cystic fibrosis (Kerem, 2004), 20%–30% in Duchenne muscular dystrophy (Chamberlain, 1997; Neri et al., 2007), and only 0.4%–1% in mucopolysaccharidosis type I (Ashton et al., 1992; Bunge et al., 1998; Oussoren et al., 2013). For most genetic diseases, the therapeutic threshold is unknown. Unlike total gene replacement, nonsense suppression approaches rely on the endogenous transcription and translation processes for target gene expression. Therefore, each clinical indication may require different PTC suppression efficiency to achieve a therapeutic threshold.

## 14 OFF‐TARGET SUP‐TRNA ACTIVITY AND TOXICITY

There has been significant concern that nonsense suppression therapeutics like sup‐tRNAs will not only suppress PTCs but also promote global readthrough and suppress NTCs. The consequence would be continued translation into the 3′‐untranslated region, resulting in C‐terminally extended polypeptides with potential toxic gain‐of‐function activities. An example of gain‐of‐function activity includes a flagrant unfolded protein response, which could result in cellular stress/toxicity (Hetz, 2012). Extended 3′ protein also has the potential to act as a novel C‐terminal antigen, however, this has not been documented. While aminoglycosides promote readthrough of nonsense codons by binding to the ribosomal decoding center and reduce translation fidelity at both nonsense and sense codons (Wangen & Green, 2020), sup‐tRNAs have not been shown to interfere with general translation fidelity, which may broaden their therapeutic window. Therefore, it is likely that any sup‐tRNA‐associated toxicity will stem from NTC readthrough.

### 14.1 Is it possible to differentiate between a PTC and a NTC?

Eukaryotic translation termination is a complex, high‐fidelity process that has been described in detailed reviews before (Dever & Green, 2012; Hellen, 2018). The naturally occurring basal NTC readthrough by misincorporation of aminoacylated‐tRNAs is as low as 0.001%–0.1% (Floquet et al., 2012; McCaughan et al., 1995; Tate et al., 1995) and 0.01%–1% at PTCs (Bonetti et al., 1995; Cassan & Rousset, 2001; Floquet et al., 2012; Manuvakhova et al., 2000), indicating a highly accurate proofreading mechanism of the translational machinery. Recent work has indicated that, in the presence of stop codon readthrough agents, stop codons in 3′‐untranslated regions are more likely to be readthrough than NTCs (Wangen & Green, 2020). The available evidence suggests that the translational apparatus can discriminate between NTCs and PTCs through distinct mechanisms (Fang et al., 2013; Nguyen et al., 2014). One factor influencing readthrough efficiency is the sequence context upstream and downstream of the stop codon [for a detailed review, see Dabrowski et al. (2015)]. Trans‐acting termination regulators play another important role in stop codon discrimination. Poly(A)‐binding proteins (PABPs), present at the 3′‐poly(A) tail of the mRNA, stimulate termination by interaction with the release factor complex (Cosson et al., 2002; Ivanov et al., 2016). The distance of the PTC from the 3′‐poly(A) tail prevents this termination enhancing interaction, resulting in ribosomal stalling at the PTC, which in turn increases the probability of PTC suppression over suppression of NTCs (Amrani et al., 2004). Of note, ribosomal stalling is also induced by UPF1, which is the key regulatory protein in NMD. While interaction between the release factor complex with PABPs promotes translation termination, interaction with UPF1 has an inhibitory effect (Ivanov et al., 2008). In the presence of a PTC, interaction of the release factor complex with UPF1 and SMG1 (SURF complex) induces NMD. NMD is an aberrant translation termination event, that leads to degradation of the PTC‐carrying mRNA (Kurosaki et al., 2019). It is strongly enhanced by downstream exon junction complex (EJC). UPF1 induced ribosomal stalling at PTCs gives enough time for EJC to interact with the SURF complex and subsequently activates NMD. Thus, the EJC downstream of the stop codon helps to discriminate between PTC and NTC (Celik et al., 2015).

With these mechanisms to differentiate between NTC and PTC in place, we predict that even at high intracellular levels of sup‐tRNA, global NTC readthrough will be infrequent. Indeed, we also looked at PTC versus NTC readthrough in the presence of ACE‐tRNAs by ribosomal profiling and observed notably more efficient readthrough of PTCs than NTCs (Lueck et al., 2019). These results are not unexpected, as NTCs face evolutionary pressure to terminate efficiently, while the genomic context of PTCs is evolved for normal translation processivity. This also suggests that a therapeutic “Goldilocks” window, where the function of the PTC‐containing gene can be restored without global disruption of translation termination at NTCs may exist (Wangen & Green, 2020).

Toxic effects of NTC readthrough may be marginal, due to safeguard mechanisms. For instance, not every C‐terminally extended protein will be toxic. Indeed, this is exemplified by numerous studies where proteins are C‐terminally tagged with GFP or epitopes. Furthermore, it is expected that C‐terminally extended proteins will be subject to the unfolded protein response before toxic accumulation (Kramarski & Arbely, 2020). However, it should be considered that even low‐level NTC readthrough of certain transcripts could result in expression of proteins with toxic gain‐of‐function. Furthermore, the aggregation and lysosomal degradation of C‐terminally extended proteins may result in a physiologically meaningful hypo‐expression of a sub‐set of proteins. Indeed, it would be interesting if nature placed additional barriers to prevent spurious NTC readthrough of transcripts whose C‐terminal extension results in toxicity. Not all stop codons exhibit equal translation termination efficiency, with the order of UGA, UAG, and UAA from “weakest” to “strongest” termination efficiency (Bonetti et al., 1995; Manuvakhova et al., 2000). Highly expressed genes often have multiple in‐frame stop codons to promote efficient translation termination, even in the presence of spurious NTC readthrough (Adachi & Cavalcanti, 2009; Keeling & Bedwell, 2011; Liang et al., 2005; Nichols, 1970; Williams et al., 2004). Both the upstream and downstream sequence context of a stop codon has a significant impact on termination efficiency (Dabrowski et al., 2015). However, a prediction of translatome NTC readthrough efficiency cannot be formulated, because the molecular mechanism for recognition of NTC sequence context remains unclear. The overall questions are, to what extent can the cell handle translatome‐wide NTC readthrough, and is there a subset of critical transcripts causing NTC readthrough toxicity? Answering these open questions will help define not only the therapeutic window for sup‐tRNAs, but also for other therapeutic readthrough technologies that have the potential to promote promiscuous NTC readthrough. One of the benefits of the sup‐tRNA technology is that any given sup‐tRNA will be specific for one of the three stop codons, therefore reducing the possibility of off‐target NTC suppression to those that do not terminate in the target stop codon (Lueck et al., 2019; Wangen & Green, 2020).

### 14.2 Outcomes of target protein expression

As previously discussed, a perceived benefit of the sup‐tRNA approach is that it relies on the endogenous transcriptional and translational regulation processes to govern expression levels and localization of target gene products, therefore eliminates the possibility of toxicity stemming from target gene overexpression. However, any therapy that results in de novo expression of endogenous gene products could result in T‐cell immunity (i.e., autoimmunity). Sup‐tRNA readthrough of disease‐causing nonsense mutations is no exception. This unwanted therapeutic outcome is highlighted in previously published studies in gene replacement studies in Duchenne muscular dystrophy patients (Mendell et al., 2010) and cystic fibrosis transmembrane conductance regulator (CFTR) knock‐out mice (Limberis et al., 2007). The impact of T‐cell immunity on outcomes of nonsense mutation therapies is not well defined. Indeed, the T‐cell response target may be dependent on the antigenicity of the therapeutic protein. This idea comes from results of several human gene replacement studies where no T‐cell immunity to the target protein was identified (Brantly et al., 2009; Cideciyan, 2010; Hauswirth et al., 2008; Maguire et al., 2008; Mendell et al., 2009). Another factor that influences the T‐cell response to rescued protein expression is the immune privilege status of the target tissue, where the eyes and central nervous system are notably immune privileged.

### 14.3 Outstanding concerns regarding therapeutic sup‐tRNAs

The two major concerns regarding the use of sup‐tRNAs as a nonsense therapeutic is toxicity stemming from NTC readthrough and overcoming the delivery hurdle. Much of our understanding of sup‐tRNA‐dependent PTC suppression is built on studies performed in prokaryotes and yeast. Results from these studies may not recapitulate all of the factors that determine sup‐tRNA activity in cultured human cells or human tissue. The use of sup‐tRNAs to readthrough disease‐causing nonsense mutations is gene therapy. A lack of delivery technologies has hampered the ability to determine the effects of PTC suppression in vivo. That is, until efficient means of sup‐tRNA delivery are developed, it is unclear what effects they will have in vivo. Another avenue yet to be explored is the generation of mice or other model organisms that express human sup‐tRNAs. This approach would allow the delivery hurdle to be circumvented to determine the impact of sup‐tRNAs on physiology in vivo. It is still not clear how much global or gene‐specific NTC readthrough is needed to result in toxicity. As previously discussed, sequence context of NTCs influences readthrough efficiency, and therefore it is likely that some transcripts are more sensitive to NTC readthrough than others. Furthermore, it is likely that not all transcripts sensitive to NTC readthrough will result in cellular toxicity when targeted. With differences in tissue gene expression profiles, there is the potential that certain tissues could be more sensitive to NTC readthrough than others. With this in mind, it may be beneficial to restrict PTC readthrough therapeutics to specific tissues. Therefore, a balance must be struck between delivery efficiency and targeted effect, for not only sup‐tRNAs, but all other PTC therapeutic gene therapy and small molecule approaches. To accelerate the use of nonsense readthrough technologies as therapeutics, including sup‐tRNAs, more information is needed on the potential impact of prolonged NTC readthrough on cellular processes.

## 15 CONCLUSIONS

The idea of using sup‐tRNAs to suppress disease‐causing PTCs was first introduced by Chang and Kan (1979) for the treatment of β^0^ thalassemia and successfully implemented shortly thereafter (Temple et al., 1982). Despite early enthusiasm, their development as a therapy has been slow to advance. Many factors have hindered the progress of sup‐tRNAs and other nonsense suppression therapy approaches. One major hurdle has been the lack of nonsense‐associated animal models of disease, where until recently most established PTC animal models were originally identified through observation of random mutations. However, the recent advancement of CRISPR/Cas technologies (Komor et al., 2017) provides the necessary means to generate small (e.g., mice) and large animal models (e.g., pigs) that harbor PTCs to test nonsense suppression agents, including sup‐tRNAs. Another hindrance is the development of nucleic acid delivery strategies. Unlike small‐molecule‐based approaches where cellular delivery is generally not considered a drawback, sup‐tRNAs are macromolecules (relatively large) that are not readily taken up by cells and therefore require effective delivery technologies. Indeed, with the development of novel delivery technologies for other RNA‐, DNA‐, and protein‐based therapies, it is reasonable to consider that efforts to develop sup‐tRNA therapeutics will also benefit from these advancements.

It is an exciting time for the development of PTC therapeutics in general. While sup‐tRNAs hold great promise, so do many other different approaches which are capable of suppressing NMD and translation termination. The end goal is to create effective PTC therapies and, likely, one approach will not be effective for all nonsense‐associated clinical indications. Therefore, it is important to promote innovative ideas and develop diverse approaches in parallel.

## CONFLICT OF INTEREST

The authors have declared no conflicts of interest for this article.

## AUTHOR CONTRIBUTIONS

**Joseph Porter:** Conceptualization; funding acquisition; writing‐original draft; writing‐review and editing. **Christina Heil:** Conceptualization; writing‐original draft; writing‐review and editing. **John Lueck:** Conceptualization; funding acquisition; writing‐original draft; writing‐review and editing.

## RELATED WIREs ARTICLES

Regulation of nonsense‐mediated mRNA decay

tRNA synthetase: tRNA aminoacylation and beyond

Controlling translation via modulation of tRNA levels

Suppression of nonsense mutations as a therapeutic approach to treat genetic diseases
